# *In Utero p*,*p*′-DDE Exposure and Infant Neurodevelopment: A Perinatal Cohort in Mexico

**DOI:** 10.1289/ehp.9566

**Published:** 2007-01-16

**Authors:** Luisa Torres-Sánchez, Stephen J. Rothenberg, Lourdes Schnaas, Mariano E. Cebrián, Erika Osorio, Maria del Carmen Hernández, Rosa M. García-Hernández, Constanza del Rio-Garcia, Mary S. Wolff, Lizbeth López-Carrillo

**Affiliations:** 1 Instituto Nacional de Salud Pública, Morelos, México; 2 Depto. Ecología Humana, CINVESTAV, Merida, Yucatán, México; 3 Instituto Nacional de Perinatología, México DF, México; 4 Sección Externa de Toxicología, CINVESTAV, México, DF, México; 5 Community Medicine, Division of Environmental and Occupational Medicine, Mount Sinai School of Medicine, New York, New York, USA

**Keywords:** Bayley scale, breast-feeding, cohort, *in utero* exposure, infant neurodevelopment, Mexico, organochlorines, *p,p′*-DDE

## Abstract

**Background:**

Evidence suggests that *p*,*p*′-dichlorodiphenyldichloroethene (DDE) affects neurodevelopment in infants, although a critical exposure window has not yet been identified.

**Objectives:**

Our goal was to assess the prenatal DDE exposure window and its effect on the psychomotor development index (PDI) and mental development index (MDI) during the first year of life.

**Methods:**

We recruited 244 children whose pregnancies and deliveries were uncomplicated, and whose mothers were monitored throughout the pregnancy. Participating mothers were not occupationally exposed to DDT (dichlorodiphenyltrichloroethane) but were residents of a zone in Mexico with endemic malaria. We measured serum levels of DDE before pregnancy and during each trimester of the pregnancy. We evaluated PDI and MDI of the Bayley Scales for Infant Development (BSID-II), at 1, 3, 6, and 12 months of age. We adjusted for quality of the home environment and maternal intellectual coefficient (IQ). We used generalized mixed-effects models for statistical analysis.

**Results:**

Third-trimester DDE level (7.8 ± 2.8 ppb) was significantly higher than the level at baseline, first, and second trimesters, but the differences never exceeded 20%. Only DDE levels during the first trimester of pregnancy were associated with a significant reduction in PDI (every doubled increase of DDE level reduced the PDI 0.5 points). DDE was not associated with MDI.

**Conclusions:**

A critical window of exposure to DDE *in utero* may be the first trimester of the pregnancy, and psychomotor development is a target of this compound. Residues of DDT metabolites may present a risk of developmental delay for years after termination of DDT use.

DDT (dichlorodiphenyltrichloroethane) is an insecticide used worldwide to combat malaria since 1942. Its principal metabolite is dichlorodiphenyldichloroethene (DDE), which, given its persistence and ubiquity in the environment, is presently found in environmental and human biologic samples (i.e., serum, maternal milk, and adipose tissue) (Turusov et al. 2002). In Mexico, DDT was used until 1999 (Yañez et al. 2004); and even recently, levels of DDE have been documented in the serum of mothers of reproductive age (Lopez-Carrillo et al. 2001; Torres-Arreola et al. 2003), in adipose tissue (Galván-Portillo et al. 2002), in maternal milk (Torres-Arreola et al. 1999), and in cord blood (Waliszewski et al. 2000) among those residents in zones both endemic and not endemic to malaria.

Levels of DDE in the fetus are similar to those found in the mother (Dorea et al. 2001), and experimental evidence shows accumulation in both the liver and brain of the fetus (Salama et al. 1993). DDT metabolites have neurotoxic capacity; they directly affect nerve cells (Klaassen et al. 1986) and have endocrine disruption effects in the hypothalamic–hypophysis–thyroid axis (Howsdeshell 2002; Takser et al. 2005). Alterations in the central nervous system have been documented concerning the visual–motor and cognitive functions of workers chronically exposed to DDT (Misra et al. 1984; Van Wendel de Joode et al. 2001). Evidence from two prospective infant cohorts shows an effect between prenatal DDE levels and hyporeflexia at 1 month of age (Rogan et al. 1986a) and damage to psychomotor and mental development at 13 months of age (Ribas-Fito et al. 2003).

Although it is possible that prenatal exposure to DDE damages early neurodevelopment, no studies have examined whether there is a critical exposure window during fetal development for disruption of subsequent development. No prior studies have measured the magnitude of the effect in the presence of other key covariables in infant neurodevelopment [such as the quality of the home environment, as measured by Home Observation for Measurement of the Environment (HOME)] (Rogan et al. 1986a; Ribas-Fito et al. 2003).

The aim of this study was to examine the prenatal window of exposure to DDE and its effect on psychomotor and mental development during the first year of life in children of mothers who are not occupationally exposed to this product.

## Materials and Methods

Between January 2001 and June 2005, 1,585 women of reproductive age, who were in the process of getting married, were invited to participate in a prospective cohort study. None of the participants had antecedents of chronic illness (thyroid, heart, liver, kidney, and/or gastrointestinal), none were treated with anticonvulsants, nor were any participants breast-feeding at the time of the recruitment. All participants were residents of four municipalities in the State of Morelos, Mexico, where DDT was used until 1998 to combat endemic malaria.

Women who refused to participate at the beginning of the study, or those who enrolled and then decided not to continue, were interviewed briefly about their age, educational level, and occupation as well as the reason for not participating.

Each participant, once informed of the objectives of the study, signed a letter of informed consent. Consent was also obtained for children once the postnatal follow-up began. This study had the approval of the Ethics Committee of the National Institute of Public Health.

### Baseline information

During the state’s obligatory prenuptial marriage counseling before the civil wedding ceremony, 1,585 eligible women were identified. Those who agreed to participate in the study were queried by previously trained interviewers about sociodemographic characteristics, gynecologic–obstetric antecedents, alcohol and tobacco consumption, occupation, as well as antecedents concerning the use of pesticides. The response rate was 62.8%.

### Prenatal follow-up

Participating women were visited every 8 weeks at their homes and/or were contacted by telephone by our research team. Once the existence of a pregnancy was confirmed by means of clinical evaluation and/or pregnancy test, an individualized home visit was planned for each of the the first, second, and third trimesters of pregnancy. The attrition rate during this stage of the study was 27.5%.

### Follow-up during pregnancy

During home visits at each trimester, a questionnaire was administered that collected data concerning the progress of the pregnancy, anthropometric measurements (maternal weight and height), and dietary information. Dietary information was obtained through a previously validated semiquantitative food frequency questionnaire (Hernandez-Avila et al. 1998). Attrition rates varied from 6.6% in the first trimester to 10.5% in the second trimester.

### Postnatal follow-up

Fifteen days before and after the estimated date of birth, calculated from the date of the last menstrual period, visits or telephone calls were made with the aim of anticipating the moment of the birth and scheduling postnatal evaluations at 1, 3, 6 and 12 months of age.

The information collected during postnatal visits varied according to the age of the child. During the visit at 1 month of age, questions related to aspects of birth were asked (gestational age, weight and size at birth, maternal and/or child complications, type of birth, and initiation of breast-feeding) and the answers were further compared with the information contained in the corresponding hospital discharge records.

During subsequent visits the information collected focused on the child’s state of health and feeding practices. At the time of the interview, the mother was asked specifically whether the child was nursing, whether breast-feeding was exclusive or combined with other food, and, if not breast-feeding, the reasons for suspending breast-feeding and introducing other foods into the diet.

Additionally, anthropometric measurements were taken (weight, length, and cephalic circumference) and neurodevelopment was evaluated for each child. Attrition rates varied from 1.5% at 3 months to 7.8% at 1 year after the birth.

### Evaluation of neurodevelopment

We used the Bayley Scales of Infant Development (BSID-II) (Bayley 1993) to evaluate the mental and psychomotor development of children during the first year of life. This test is valid for children between 1 and 42 months of age, and consists of two indices: one that evaluates mental development (MDI) and the other, psychomotor development (PDI). We applied a Spanish version, used in previous studies concerning neurodevelopment and environmental exposure to lead in Mexico (Gomaa et al. 2005). Two trained psychologists, blinded to maternal levels of DDE during the pregnancy, administered the BSID-II test. The interobserver concordance was 0.96 for MDI and 0.98 for PDI.

### Maternal intelligence

The maternal intellectual coefficient (IQ) was measured using a Spanish version of Wechsler Adult Intelligence Scale (Wechsler 1981).

### Evaluation of the quality of the environment and home

When the children were 6 months of age, we administered a Spanish version of the HOME Scale (Home Observation for Measurement of the Environment) (Caldwell and Bradley 1984). This instrument evaluated the quality of the home environment as a determinant of neurodevelopment and as a possible confounder in neurotoxic studies. The test covered parent–child communication and interaction, the type of toys available, specific events and other experiences that occur in the home, as well as the general and physical quality of the home environment. The total score of this test was used in the analyses.

### Chemical analysis

#### DDT metabolites

Blood (7 mL) was taken from each of the participants during the baseline interview and/or at each trimester visit. After centrifugation, the serum obtained was stored at −70°C in glass vials (prewashed with pesticide hexane grade) covered with a Teflon cap, until it was analyzed.

We determined the levels of DDE and *p,p*′-DDT in serum by means of gas chromatography with an electron capture detector (model 3400; Varian, Inc., Palo Alto, CA, USA), following the protocol recommended by the U.S. Environmental Protection Agency (1980). Concentrations of DDE and *p,p*′-DDT were reported in wet basis as nanograms per milliliter (parts per billion). The detection limit was 0.05 ng/mL and 0.0045 ng/mL for DDE and *p,p*′-DDT, respectively. All (100%) of the serum samples were positive for DDE, whereas only 11–22% (depending on the trimester of pregnancy) were positive for *p,p*′-DDT.

For internal quality control, each of the serum samples was fortified with aldrin and the average recovery was 98.15 ± 8.8%. For every 10 study samples, one sample of bovine serum with known quantities of β-hexachlorocyclo-hexane (β-HCH), aldrin, hexachlorobenzene (HCB), DDE, and 1,1-dichloro-2,2-bis(*p*-chorophenyl)ethane (*p,p*′-DDD) was analyzed, with recovery of 100.8, 100.01, 100.91, 103.4, 104.1%, respectively. Additionally, one randomly selected sample was analyzed in duplicate in each batch with a coefficient of variation of 4.37% and 0.45% for DDE and *p,p*′-DDT, respectively. The results of the external quality control comparing our laboratory (CINVES-TAV) and M. Wolff’s laboratory in the Division of Environmental and Occupational Medicine at Mount Sinai Medical School showed a coefficient of Bland-Altman correlation between 10 split serum samples for DDE of 0.98.

#### Lead analysis

Maternal blood was collected in trace metal-free tubes at each trimester of pregnancy. Blood lead levels were determined in duplicate analysis in ESA laboratories (Environmental Science Associates Laboratories, Inc., Chelmsford, MA, USA), using a voltammetric anodic separation method. Samples with mean levels < 5 μg/dL were reanalyzed using atomic absorption spectrometry (model 3000; Perkin-Elmer, Inc., Norwalk, CT, USA). External quality control samples were provided by Centers for Disease Control and Prevention laboratories (Atlanta, GA, USA) and Pennsylvania State Blood Lead Proficiency Testing Program (Exton, PA, USA).

### Statistical analysis

Because distributions of residuals of the regressions of Bayley indices on concentrations of DDE in each trimester of the pregnancy were right-skewed and there was evidence of heteroskedasticity, we natural log-transformed DDE. To facilitate interpretation of the regression coefficients of DDE, the change of PDI or MDI for each two-times increase of DDE was calculated from the natural log coefficients in the models by transforming those coefficients to log base 2 DDE by multiplying the original natural log coefficients by 0.69.

To evaluate the effect of exposure to DDE in each trimester and other covariates on psychomotor (PDI) and mental development (MDI) of the child during the first year of life, we used generalized mixed effects models, as specified in the following equation:


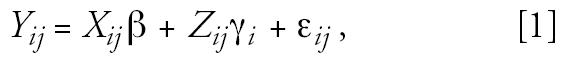


where *ij* represents the observation *j* in the subject *i; Y_ij_* corresponds to the dependent variables mental or psychomotor development of each subject during the first year of life (1, 3, 6, and 12 months respectively), and *X_i_* are the independent variables with fixed effects: levels of DDE at each trimester of pregnancy (on a logarithm scales), age (years), education (years), body mass index (BMI) at the beginning of pregnancy (kilograms per square meter), maternal IQ, history of hypertension induced by the pregnancy (yes/no), type of birth (vaginal/cesarean), sex of child (female/male), weight at birth (grams), breast-feeding (yes/no) at each evaluation (1, 3, 6, and 12 months), age at each evaluation, scale of quality of home environment (HOME Scale) at 6 months of age, and the education level of father (years). We also evaluated the effect of DDE at each stage of pregnancy with separate models, in which the independent variable of interest corresponded to the concentration of DDE in maternal serum for pre-pregnancy and the corresponding trimester being studied.

Further, *Z_ij_*γ are the variables with random effects. The model assumes that the random effects have a normal distribution, with the average equaling 0 and with constant variation. To determine whether the addition of a random intercept significantly improved the adjustment of the model, we used the test for maximum likelihood with an α < 0.05.

Finally, for *i*, in PDI models we also included random slopes of age at evaluation and age-squared to account for the nonlinear change in group performance with age. In MDI models we included only a linear random slope of age at evaluation. We assumed an unstructured covariance.

We explored a possible modification of the effect of DDE on neurodevelopment caused by breast-feeding, incorporating a term for the interaction between both variables into the model. A probability value ≤ 0.05 for the interaction was considered statistically significant.

The diagnosis of the model consisted of the estimation of residuals by subtraction of the recorded values of PDI and MDI from the model linear prediction of those values. We also used the Shapiro-Wilks and Shapiro-Francia tests and an evaluation using histograms and normal quantile graphs to evaluate residual normality. Graphs of model prediction versus standardized residuals permitted evaluation of residual homoskedasticity.

All analyses were made using STATA 9.2. (StataCorp., College Station, TX, USA)

## Results

**Figure 1:**
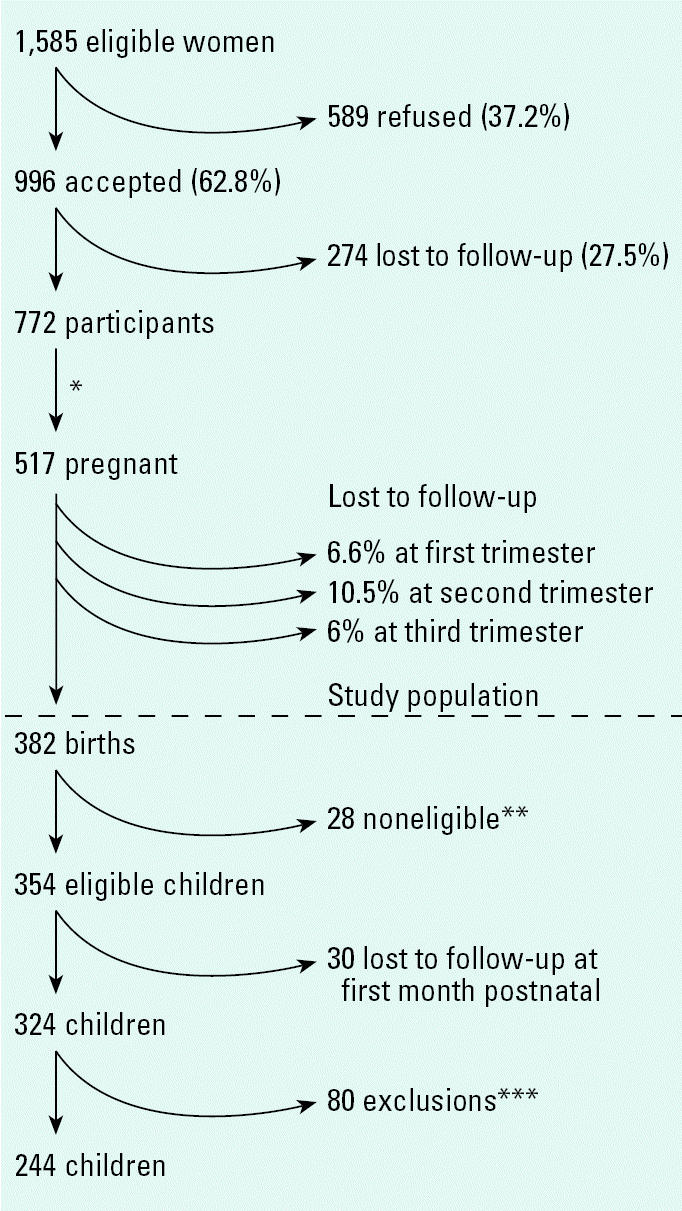
Prospective cohort study in Morelos, Mexico, 2001–2005: recruitment flow chart. *Average time between study recruitment and time to pregnancy: 8 months. **Four neonatal deaths, six children outcome of twin pregnancies, one child born at 25 weeks, one child diagnosed with congenital hypothyroidism, one child diagnosed with severe cerebral atrophy, and 15 children diagnosed with with perinatal asphyxia and/or birth weight < 2 kg and maternal age ≤ 15 years. ***Only one Bayley test and/or no DDE maternal levels during pregnancy.

As shown in Figure 1, a total of 1,585 eligible women were identified, of whom 996 (62.8%) agreed to participate in the cohort. When compared with those who did not agree to participate (589), the cohort was significantly younger (22.6 vs. 23.8 years), had a lower educational level (11 vs. 12.3 years), and a higher proportion of women were dedicated to household activities (51.3 vs. 42.8%) (data not shown). Among 517 pregnancies recorded in the cohort, 382 births had taken place at the time of this report. Between pregnant women who remained in the study and those who were lost to follow-up, no significant differences (*p* > 0.05) were found in age (21.9 vs. 21.7 years), education level (10.7 vs. 10.4 years), occupation (45.6 vs. 44%), and parity > 1 (18.9 vs. 14.7%) (data not shown). The principal reasons for dropping out of the study were a lack of interest and change in address.

Out of 382 births, a total of 28 noneligible children were identified: four neonatal deaths, six children who were outcomes of twin pregnancies, one child who was premature at 25 weeks, one who was diagnosed with congenital hypothyroidism, and another with severe cerebral atrophy. Additionally, 15 children were excluded for perinatal asphyxia diagnosis and/or a birth weight of < 2 kg and a maternal age ≤ 15 years. Eighty children were excluded in further analysis because they had only one postnatal evaluation and/or they lacked data for maternal serum levels of DDE; the final study population comprised 244 children.

In relation to maternal age, education, paid occupation, and parity > 1, we found no significant differences among children included in the analysis, exclusions, and those who were lost to follow up (Table 1). Selected characteristics of the study population are described in Table 2; third-trimester DDE level (7.8 ± 2.8 ppb) was significantly higher than in the baseline period and first and second trimesters, but the differences never exceeded 20% (analyses not shown).

Bayley MDI and PDI scores at each age are reported in Table 3. The scores obtained for MDI and PDI were > 90 at 1, 6, and 12 months of age. Table 4 shows that higher DDE levels in the first trimester of pregnancy are significantly associated with reduced PDI of the child during the first year of life. After adjusting for breast-feeding, birth weight, age at evaluation, and HOME, a reduction of 0.52 points in PDI was associated with every doubling of DDE level (equivalent to a reduction of ~ 2.0 points for each 10-fold increase in DDE levels). We found no significant association between neurodevelopment and maternal DDE levels at the second or third trimester. Likewise, PDI and MDI group performances were significantly greater at 1 month of age compared with the other three evaluations conducted during the first year of life, regardless of DDE levels.

Although children who were breast-fed had higher PDI and MDI scores than children who were not breast-fed, this effect was statistically significant only for MDI [1.14 points; 95% confidence interval (CI), 0.08–2.20), independent of DDE level. Both HOME scale and birth weight were associated with a significant increase in PDI and MDI, independent of exposure to DDE. A difference of one point in the HOME scale index for measurements of home environment was associated with an increase in PDI of β = 0.24 (95% CI, 0.10–0.40) and an increase in MDI of β = 0.14 (95% CI, 0.04–0.24). Each additional kilogram in birth weight was associated with an increase of β = 2.60 (95% CI, 1.04–4.20) and β = 2.14 (95% CI, 1.07–3.21) for PDI and MDI, respectively.

The interaction between current maternal breast-feeding and exposure to DDE during the first trimester was not statistically significant (β = 0.62; interaction *p* = 0.20) (data not shown). There was no significant interaction between the HOME Scale and DDE with regard to PDI (β = −0.02; interaction *p* = 0.76) or to MDI (β = −0.03; interaction *p* = 0·60) (data not shown).

Finally, geometric mean lead levels during all trimesters of pregnancy in a random sub-sample (*n* = 105) were between 5.5 ± 1.8 μg/dL at first trimester and 5.6 ± 1.9 μg/dL at third trimester, with a maximum of 27 μg/dL. The correlation between lead levels and DDE was −0.11 (*p* = 0.02), and the respective coefficients for DDE with PDI and MDI with and without adjusting for lead were β = −0.52 versus −0.48 for the PDI and −0.18 versus −0.12 for the MDI, respectively. The estimates for the remaining trimesters did not appear to be affected by the lack of adjustment for lead (data not shown). There was no interaction between the lead levels and DDE in relation to PDI (data not shown).

## Discussion

The results of this study suggest that fetal exposure to DDE during the first trimester of pregnancy may negatively affect psychomotor development of the child during the first year of life. Additionally, we observed a protective effect of breast-feeding on mental development (Anderson et al. 1999). Increased birth weight and quality of the home environment (HOME) were associated with increased PDI and MDI over the first 12 months of life.

Other studies have reported harmful effects of exposure to DDT during pregnancy. Using the Bayley Scale, Ribas-Fitó et al. (2003) reported a decrease of 4.01 points (95% CI, −6.7 to −1.3) at 13 months of age in PDI for each doubling of DDE measured in umbilical serum, which is greater than the −0.52-point (95% CI, −0.96 to −0.075) decrease associated with each doubling of first trimester DDE (~ 2 points for each 10-fold increase in DDE levels) reported in this study. They also identified a similar negative effect caused by DDE on MDI (3.5 points), whereas in this study we failed to detect a significant DDE effect during any trimester on MDI. Their study did not control for the HOME Scale. The lack of adjustment for HOME Scale in studies of lead exposure results in an overestimation of 4–5 points of lead effect on the IQ of the child (Mink et al. 2004).

In our study, the associations between the HOME Scale and the two areas of development evaluated were statistically significant (*r_p_*= 0.15, *p* = 0.000 for PDI; 0.16, *p* = 0.000 for MDI) and the correlation coefficient between the Home Scale and DDE levels was −0.19 (*p* = 0.000), suggesting that the children who were most exposed to this compound *in utero* were those who also had a less stimulating developmental environment. Thus the DDE coefficient associated with PDI (β = −0.60; 95% CI, −0.99 to −0.17) with no adjustment by HOME scale was 15% greater than the coefficient associated with PDI (β = −0.52) when the HOME variable was included in the final model. However, for the MDI the difference observed between the crude and adjusted coefficient was much greater (β = −0.08 vs. β = −0.06), a difference of almost 30%. Consequently, lack of control for the HOME Scale might explain part of the higher estimated DDE coefficients in previous studies (e.g., Ribas-Fito et al. 2003).

In another cohort study, no association was found between prenatal exposure to DDE and either motor or mental development at 1 year of age (Gladen et al. 1988). Also, in contrast to the previous study, where a mixture of matrices (umbilical cord, maternal serum, placenta) were used to estimate fetal DDE exposure (Gladen et al. 1988; Rogan et al. 1986b), we used maternal serum samples at each trimester of pregnancy, a more valid estimation of such exposure (Dorea et al. 2001).

In our 1999 pilot study, we found no evidence of the presence of other potential confounders, such as PCBs, arsenic, or organo-phosphates pesticide exposure, in the study area (unpublished data), but we did identify an average value for blood lead levels of 4.6 μg/dL (Moline et al. 2000). We evaluated its impact in a random subsample of 105 mothers of this study, and results suggest that not including the lead levels in our analysis may have caused a slight underestimate of the effect of DDE on neurodevelopment during the first trimester of pregnancy.

To determine whether the effect of first-trimester DDE on the PMI varied as a function of child’s age from 1 to 12 months, we constructed an alternative model testing interactions of DDE on the PMI at each of the four age periods. None of the interactions at 3, 6, or 12 months were significantly different from the DDE effect at 1 month (*p* > 0.52), nor were the 3- to 12-month interactions significantly different among themselves (*p* > 0.66), nor were the 3- to 12-month interactions considered jointly different from the effect at 1 month (*p* > 0.57); and the model with interactions represented no significant improvement in fit to the data than the model without interactions (*p* > 0.90). We conclude, within the limitations of the power of our data set, that there were no detectable differences in DDE effect on PMI from 1 to 12 months of age.

Other potential confounders include the levels of hemoglobin in blood during the first year of life. According to recent studies, 15% of children < 1 year of age may have levels of hemoglobin < 95 g/L (Rivera Dommarco et al. 2001), which could have a negative impact on MDI (Kordas et al. 2004) and possibly on PDI (Beard and Connor 2003). No information is available to explore the relationship between the levels of DDE and the concentration of hemoglobin in this population. However, the expected prevalence of childhood anemia is low in children < 1 year of age, which makes it unlikely that the reported results have been considerably distorted. The same consideration can be made concerning the low prevalence in this population of either maternal smokers (8%) or those consuming alcohol during pregnancy (5%).

To compare our results with those of previous studies, we reported nonlipid corrected DDE values. However, we must acknowledge that correcting by lipids produced essentially the same association between DDE levels during first trimester of pregnancy and PDI (βDDE lipid = −0.38; *p* = 0.08).

The high interlaboratory concordance coefficient of DDE serum values suggests valid estimates. In contrast, the possibility that a differential measurement error might have distorted our results is not very likely, because the psychologists who evaluated neurodevelopment did not know the maternal levels of DDE, nor did the laboratory technicians know of the neurodevelopment of the children.

We did not find significant effects of prepregnancy DDE levels on subsequent child development. Our findings show that the critical window of exposure to DDE may be the first trimester of pregnancy and that the damage appears in motor development. The first trimester of pregnancy is critical for the central nervous system and neuron development (Rice and Barone 2000). Cerebral development is a sequential and limiting process, the magnitude of the damage being greater the earlier it occurs (Rice and Barone 2000). The possible mechanisms of neurologic damage that involve DDT and/or its metabolites are a direct effect on motor fibers and on the motor area of the cerebral cortex and their effect as endocrine disruptors in the hypothalamic–hypophysis–thyroid axis.

To demonstrate the effect on the cerebral cortex, animal experiments (Eriksson et al. 1992) have shown a significant reduction in the density of muscarinic cholinergic receptors as a result of DDT neonatal exposure. These receptors are predominantly cholinergic and are directly involved in the process of neuronal excitement and inhibition (Eriksson et al. 1992). A second mechanism, and probably the most widely accepted for explaining the chronic effect of DDT, is disruption of the thyroid system (Takser et al. 2005). DDT, whether directly or through its metabolites, alters the production of thyroidal hormone and hormone availability at target tissues, given that DDT blocks glucuronidation. It is also known that thyroid hormone plays an important role in cerebral development and serves as a signal for neuronal differentiation and maturation (Anderson et al. 2003). Studies have demonstrated that thyroid hormone participates in neuronal migration and proliferation (Lavado-Autric et al. 2003) as well as synaptogenesis and myelinization, all processes that begin during the first trimester of pregnancy (Rice and Barone 2000).

The size of the effect of exposure to DDT on child development is similar to that reported for lead; over the 10th–90th percentile range in levels of DDE exposure during the first trimester of pregnancy (1.4–18.8 ng/mL) we observed a reduction in the psychomotor development of ~ 4 points. Other investigators found a difference of 4 points in mental development for lead-exposed children < 2 years of age (Bellinger et al. 1988), where children from the lowest tertile of exposure to this metal were compared with those in the highest tertile (≤ 3 vs. > 10 μg/dL).

The growing evidence that metabolic by-products of DDT present in the mother during pregnancy impede infant psychomotor development over the first 12 months of life, combined with the long residence times of the metabolites in the environment and in the human body, suggests the need for increased vigilance of prenatal exposure, even in countries that suspended DDT use years and even decades ago.

## Figures and Tables

**Table 1:** Selected maternal characteristics of participating/nonparticipating children.

Characteristic	Included in the analysis (*n* = 244)	Exclusions (*n* = 80)	Lost to follow-up (*n* = 30)
Age [years (mean ± SD)]	21.7 ± 3.9	22.3 ± 4.2	22.1 ± 4.6
Education [years (mean ± SD)]	10.6 ± 3.1	11.3 ± 3.4	9.8 ± 2.8
Paid occupation (%)	44.7	53.7	43.3
Parity > 1 (%)	18.4	17.5	20.0

Statistical tests used were *t*-test for age, education, and birth-weight, and chi-square for occupation and parity.

**Table 2:** Selected characteristics of the study population.

		Percentiles	
Variable	*n* = 244	10th	90th
Maternal			
Intellectual coefficient (mean ± SD)	86.7 ± 12.3	71	102
BMI (mean ± SD)^a^	23.2 ± 3.7	18.7	28.6
Pregnancy-induced hypertension (%)	7.0		
Percent cesarean birth	55.3		
Child			
Percent male	56.7		
Birth weight [g (mean ± SD)]	3,241 ± 428.8	2,735	3,850
Breast-feeding (%)			
Not breast-fed	7.8		
≤ 12 weeks	26.2		
> 12 weeks	66.0		
HOME Scale (mean ± SD)	30.1 ± 4.6	25	36
*p,p*′-DDE maternal serum levels [ng/mL, GM (GSD)]			
Prepregnancy (*n* = 174)	6.8 (2.8)	1.7	21.8
1st trimester (*n* = 213)	6.4 (2.8)	1.4	18.8
2nd trimester (*n* = 172)	6.8 (2.9)	1.3	23.6
3rd trimester (*n* = 181)	7.8 (2.8)	1.8	26.3

Abbreviations: GM, geometric mean; GSD, geometric SD.

a At first trimester of pregnancy.

**Table 3:** Distribution of PDI and MDI in the Bayley Scale according to age at evaluation (mean ± SD).

Age at evaluation (months)	PDI	MDI
1 (*n* = 225)	97.6 ± 7.2	98.6 ± 4.5
3 (*n* = 233)	86.4 ± 4.1	94.7 ± 5.5
6 (*n* = 227)	94.8 ± 8.7	96.3 ± 4.2
12 (*n* = 191)	91.0 ± 7.4	94.1 ± 7.1

**Table 4:** Mixed-effects models^a^ for the impact of prenatal exposure to *p,p*′-DDE on infant psychomotor and mental development throughout the first year of life.

	PDI			MDI		
Variable	Coefficient	95% CI	*p*-Value	Coefficient	95%CI	*p*-Value
*p,p*′-DDE (ng/mL)^b^						
1st trimester	− 0.52^c^	−0.96 to −0.075	0.02	−0.06^c^	−0.36 to 0.24	0.69
2nd trimester	0.23^c^	−0.22 to 0.69	0.32	−0.12^c^	−0.43 to 0.20	0.47
3rd trimester	0.16^c^	−0.30 to 0.62	0.50	0.07^c^	−0.24 to 0.40	0.64
Age at evaluation (months)^d^						
3	−10.80	−12.10 to −9.45	0.000	−2.59	−3.55 to −1.63	0.000
6	−1.94	−3.46 to −0.42	0.01	−1.24	−2.24 to −0.24	0.01
12	−5.72	−7.46 to −3.98	0.000	−3.30	−4.67 to −1.91	0.000
Breast-feeding (yes)	1.14	−0.34 to 2.62	0.13	1.14	0.08 to 2.20	0.03
HOME Scale	0.24	0.10 to 0.40	0.001	0.14	0.04 to 0.24	0.005
Birth weight (kg)	2.60	1.04 to 4.20	0.001	2.14	1.07 to 3.21	0.000

a Generalized mixed models effects, with the subject as a random intercept and random slopes of age at evaluation and age-squared for PDI models. MDI models only included subject as a random intercept and random slope of age at evaluation. Models were adjusted for birth weight, age at evaluation, breast-feeding, and HOME Scale at 6 months of age.

b log_2_DDE.

c For each doubling of *p,p*′-DDE.

d Compared with evaluation at 1 month of age.
